# KSHV-induced ligand mediated activation of PDGF receptor-alpha drives Kaposi's sarcomagenesis

**DOI:** 10.1371/journal.ppat.1007175

**Published:** 2018-07-09

**Authors:** Lucas E. Cavallin, Qi Ma, Julian Naipauer, Sachin Gupta, Mani Kurian, Paola Locatelli, Paolo Romanelli, Mehrdad Nadji, Pascal J. Goldschmidt-Clermont, Enrique A. Mesri

**Affiliations:** 1 Viral Oncology Program, Sylvester Comprehensive Cancer Center, Miami Center for AIDS Research, Department of Microbiology & Immunology, University of Miami, Miami, Florida, United States of America; 2 Department of Dermatology, University of Miami, Miami, Florida, United States of America; 3 Department of Pathology, University of Miami, Miami, Florida, United States of America; 4 Division of Cardiology, Department of Medicine, University of Miami Miller School of Medicine, Miami, Florida, United States of America; University of North Carolina at Chapel Hill, UNITED STATES

## Abstract

Kaposi’s sarcoma (KS) herpesvirus (KSHV) causes KS, an angiogenic AIDS-associated spindle-cell neoplasm, by activating host oncogenic signaling cascades through autocrine and paracrine mechanisms. Tyrosine kinase receptor (RTK) proteomic arrays, identified PDGF receptor-alpha (PDGFRA) as the predominantly-activated RTK in KSHV-induced mouse KS-tumors. We show that: 1) KSHV lytic replication and the vGPCR can activate PDGFRA through upregulation of its ligands PDGFA/B, which increase c-myc, VEGF and KSHV gene expression in infected cells 2) KSHV infected spindle cells of most AIDS-KS lesions display robust phospho-PDGFRA staining 3) blocking PDGFRA-signaling with N-acetyl-cysteine, RTK-inhibitors Imatinib and Sunitinib, or dominant-negative PDGFRA inhibits tumorigenesis 4) PDGFRA D842V activating-mutation confers resistance to Imatinib in mouse-KS tumorigenesis. Our data show that KSHV usurps sarcomagenic PDGFRA signaling to drive KS. This and the fact that PDGFRA drives non-viral sarcomas highlights the importance for KSHV-induced ligand-mediated activation of PDGFRA in KS sarcomagenesis and shows that this oncogenic axis could be successfully blocked to impede KS tumor growth.

## Author summary

Signaling mimicry is a key mechanism whereby oncoviruses can usurp host-regulatory pathways leading to acquisition of tissue-specific cancer hallmarks. A critical question in the KS field is the identification of this host pathways activated by KSHV that could provide novel insights on KSHV-pathobiology, elucidating new druggable pathways. Here we show that KSHV lytic replication as well as the KSHV-oncogene vGPCR activates PDGFRA signaling through upregulation of its ligands PDGFA/B, and that blocking of PDGFRA signaling is anti-tumorigenic. This indicates that approaches that fully and stably inhibit PDGFR-signaling could lead to successful treatments for KS, validating this receptor-ligand signaling-axis as a therapeutic target.

## Introduction

Kaposi’s sarcoma herpesvirus (KSHV) is the etiological agent of Kaposi’s sarcoma (KS) [1–4]. KS is a major cancer associated with AIDS (AIDS-KS) and is the most prevalent type of cancer affecting men and children in Africa [2, 3, 5]. It is characterized by spindle cell proliferation, intense angiogenesis and erythrocyte extravasation with variable inflammatory infiltrates [2, 3]. Although the incidence of AIDS-KS in the western world has markedly declined since the wide-spread implementation of HAART, a significant percentage of AIDS-KS patients never achieve total remission [5–7]. Moreover, KSHV prevalence and KS incidence appear to be increasing, even in HAART treated HIV patients with controlled viremias [8, 9]. Understanding the interplay of viral and host factors in KS oncogenesis is critical for the rational development of new therapies [10, 11]. Many host signaling cascades co-opted by KSHV including PI3K/AKT/mTORC, NFkB and Notch are critical for cell-specific mechanisms of transformation and their identification is paving the way to therapeutic target discovery [4, 12–15].

KSHV-infected KS lesions are composed of latently-infected cells, as well as cells expressing lytic genes that have been implicated in the development of the KS angioproliferative phenotype via paracrine and autocrine mechanisms [2–4, 16–18]. These mechanisms are mediated by angiogenic lytic viral genes such as the G protein-coupled receptor (vGPCR) homologous to the human chemokine receptors CXCR1/2 [16], K1 and K15. The vGPCR oncogene is able to induce KS-like tumorigenesis in transgenic mice [19, 20], and it is essential for angiogenesis and tumorigenesis in the mECK36 KSHV-induced tumor mouse model [21]. vGPCR activates MAPK, PI3K/AKT and NFkB signaling leading to the secretion of angiogenic and inflammatory factors most notably, VEGF and PDGF [16, 18, 22], which are both KS spindle cell growth factors. Yet, anti-VEGF interventions had limited success in KS [23] suggesting that other important paracrine mediators may also play a critical role. Anti-PDGFR approaches such as Imatinib, have shielded more promising clinical effects in AIDS-KS suggesting that this pathway might be more central to KS pathogenesis. Analysis of the molecular KS signature common to human KS tumors and our mouse KS-like tumors, showed consistent expression of KS markers VEGFR 1, 2, 3, Podoplanin with upregulation of angiogenesis ligands and receptors *in vivo*, pointing to the upregulation of various receptor tyrosine kinase signaling axes [3, 21]. Thus, we set out to rank the host tyrosine kinase signaling cascades activated by KSHV using the mECK36 model of KSHV-dependent tumorigenesis. We found that PDGFRA is the most activated RTK in mouse-KS and show that KSHV lytic genes including the KSHV vGPCR oncogene can activate it by up-regulating PDGFs. We show that PDGFRA is a driver of KSHV-tumorigenesis, and show that PDGFRA is prominently activated in murine and human AIDS-KS. Finally we show that fully blocking PDGFRA signaling impedes murine KS tumor formation.

## Results

### Proteomic analysis of receptor tyrosine kinases in mouse KSHV-induced KS-like tumors shows activation of PDGF receptor alpha

**Fig 1:**
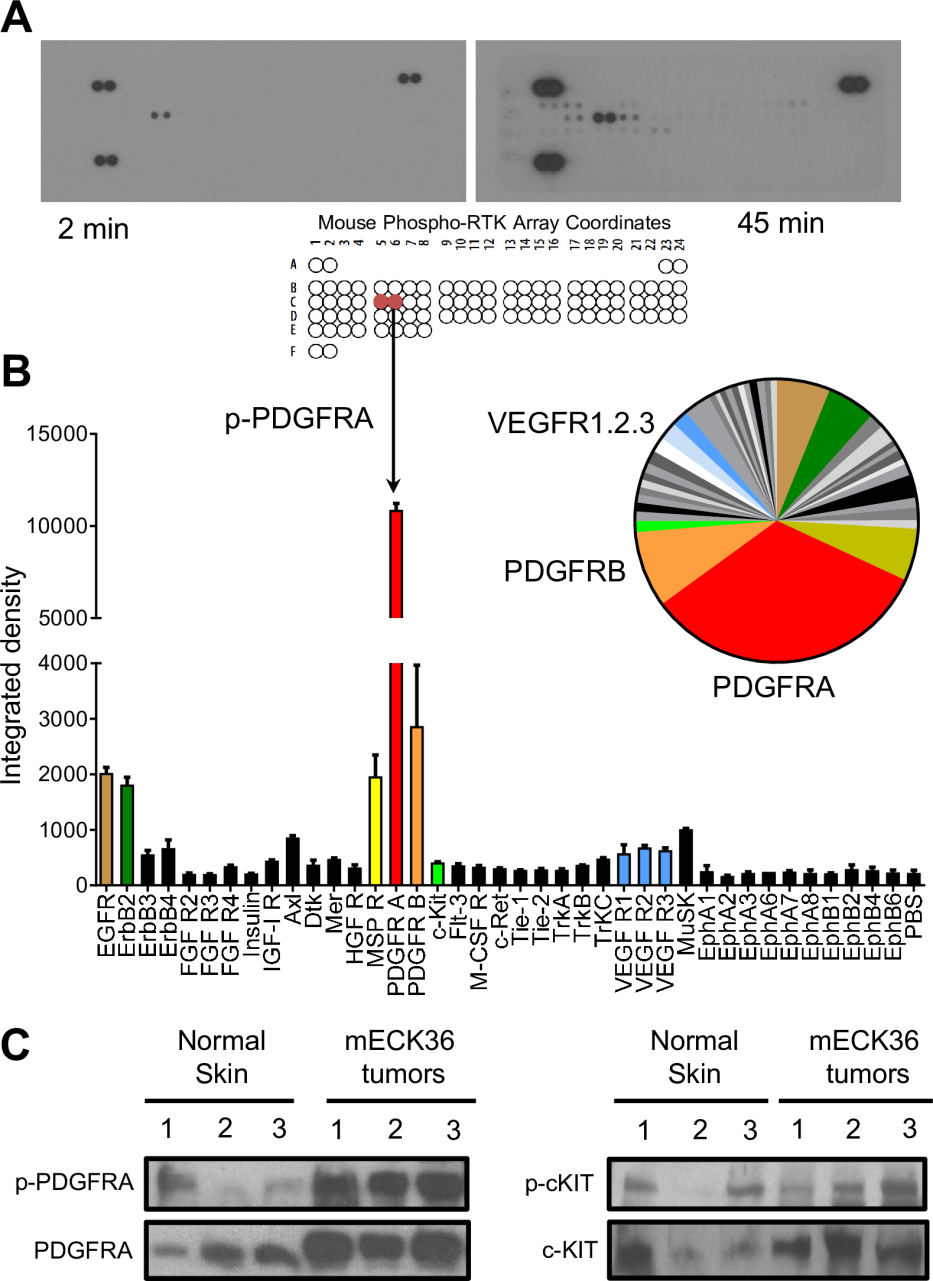
Proteomic analysis of receptor tyrosine kinases in mouse KSHV-induced KS-like tumors shows activation of PDGF receptor-alpha. (**A**) Mouse Phospho-Receptor Tyrosine Kinase (RTK) Array Kit used to quantify levels of phosphorylation of 39 RTKs in mECK36 tumors pointing to major activation spot corresponding to PDGF receptor alpha chain. (**B**) Bar graph and pie chart from densitometry for the higher-exposure blot are equally color coded for the most prominent signals. (**C**) PDGFRA and phospho-PDGFRA (left panel) or c-KIT and phospho-cKIT (right panel) determined in 3 different samples of Mouse Normal Skin and mECK36 tumors from 3 different mice by immunoblotting.

Mouse bone-marrow endothelial-lineage cells (mEC) transfected with the KSHVBac36 (mECK36) form KSHV-infected tumors in nude mice thus providing a platform to dissect molecular mechanisms of tumorigenesis by KSHV [21]. mECK36 tumor formation show consistent expression of the KS markers Podoplanin and LYVE-1, occurring with concomitant up-regulation of KSHV lytic oncogenes and angiogenesis ligands/ receptors, pointing to the upregulation of various receptor tyrosine kinase signaling axes[21]. To rank the tyrosine kinase receptors most activated in mECK36 KSHV-induced KS-like tumors [21] in an unbiased manner, we used a tyrosine kinase proteomic array (Fig 1A and 1B). Surprisingly, analysis of mECK36 tumor tyrosine kinase activation (Fig 1A) showed a prominently activated RTK spot that accounted for a significant portion of the total tyrosine kinase activation of the tumor (Fig 1B), which corresponded to the PDGF receptor alpha-chain (PDGFRA). PDGFRB was also activated, although 4 times less than PDGFRA. Other RTKs such as Axl, EGFR, MSPR and ERBB2 that have been described as implicated in KS and other cancers, were also activated; yet at a lesser extent than PDGFRA (Fig 1A)[24–26]. The receptors for the VEGF family VEGFR1/2, which are strongly expressed by mECK36 tumors [21], were also phosphorylated as expected, albeit at lower levels. This data is consistent with the predictions from the analysis of a KS- signature derived from differential expressed genes between KS and normal skin, which predicted the activation of several paracrine axes by upregulation of receptors and/or their ligand [3]. The western blots of Fig 1C depict the level of expression and phosphorylation for PDGFRA (left panel) and c-kit (right panel), a related RTK that does not display high levels of activation in the array for mECK36 tumors (Fig 1A and 1B). As a normal tissue control, we used normal mouse skin, a control tissue that has been used for generating KS signatures. Fig 1C shows that PDGFRA is robustly expressed and much more phosphorylated in the tumors than in skin control. On the other hand, c-kit is similarly expressed and displays low levels of activation in the tumors and in the skin control. Such robust expression and activation for a RTK in a tumor proteomic array is typical of an oncogenic driver. In fact, activation of PDGFRA has been described as a driver of oncogenic signaling in several non-viral sarcomas such as Gastrointestinal Stromal Tumors (GIST) and synovial sarcomas [27, 28]. Noteworthy, PDGF ligands and receptors are expressed in AIDS-KS lesions [29, 30], can induce KS spindle cell proliferation [31, 32], and are a feature of the KS and molecular mECK36 signatures indicative of potential paracrine and/or autocrine activation of PDGFRA [3, 21]. Taking together, this and our data supports the possibility that PDGFRA activation could be driving an oncogenic signaling axis for KS as suggested by recent Imatinib AIDS-KS trials [33, 34].

### Phosphorylation of PDGFRA in mECK36 and KS tumors is associated with the presence of its PDGF ligands and appear in areas of KSHV infection

**Fig 2:**
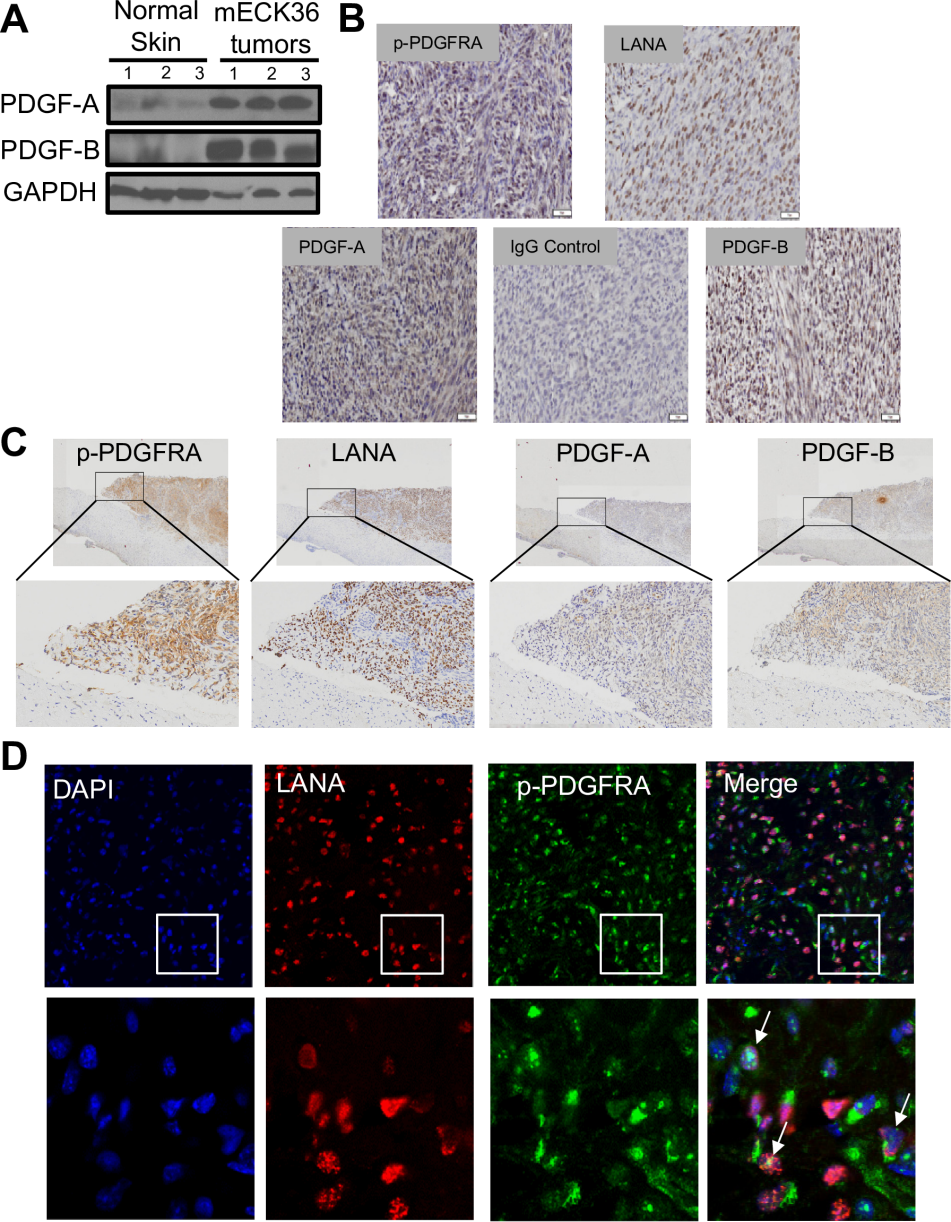
Phosphorylation of PDGFRA in mECK36 and KS tumors is associated with the presence of its PDGF ligands and localize to areas of KSHV infection. (**A**) PDGFA, PDGFB and GAPDH determined in 3 different samples of Normal Skin and mECK36 tumors from 3 different mice by immunoblotting. (**B**) Immunohistochemical staining of mouse KS-like mECK36 tumors for PDGFA, PDGFB, LANA, and phospho-PDGFRA. Representative image of the FFPEs obtained from the mECK36 tumorigenesis experiment N = 5.(**C**) Immunohistochemical staining of human KS biopsies for PDGFA, PDGFB, LANA, and phospho-PDGFRA. (**D**) IFA of mECK36 tumors for KSHV LANA (red) and phospho-PDGFRA (green). Cell nuclei were counterstained with DAPI (blue). White arrows indicate cells that co-stain for LANA and p-PDGFRA.

Due to the prominent PDGFRA phosphorylation that we observed and its potential driver-role in KS, we sought to identify KSHV-driven mechanisms of PDGFRA activation. KSHV encodes many viral oncogenes with potential to upregulate PDGF expression leading to ligand-mediated PDGFRA activation. In fact, compared to a control uninfected tissue, mECK36 tumors display very high levels of the PDGFRA ligands PDGFA and PDGFB (Fig 2A). To determine whether a KSHV-linked mechanism of PDGF upregulation was responsible for PDGFRA phosphorylation in mECK36 tumors and AIDS-KS lesions, we carried out an immunohistochemical analysis for phospho-PDGFRA, KSHV-LANA (latency-associated nuclear antigen, a marker of KSHV infection), PDGFA and PDGFB. We found that KSHV-LANA staining appears in areas corresponding to phosphorylated PDGFRA, as well as, diffuse PDGFA and PDGFB expression in mECK36 tumors and in AIDS-KS lesions (Fig 2B and 2C). To better visualize phospo-PDGFRA and LANA localization in mECK36 tumors we performed double staining immunofluorescence analysis (IFA). As shown in Fig 2D phospho-PDGFRA co-distributed with KSHV LANA supporting a link between KSHV infection and PDGFRA activation.

### KSHV Lytic switch *in vivo* and *in vitro* induces PDGF-mediated PDGFRA activation

**Fig 3:**
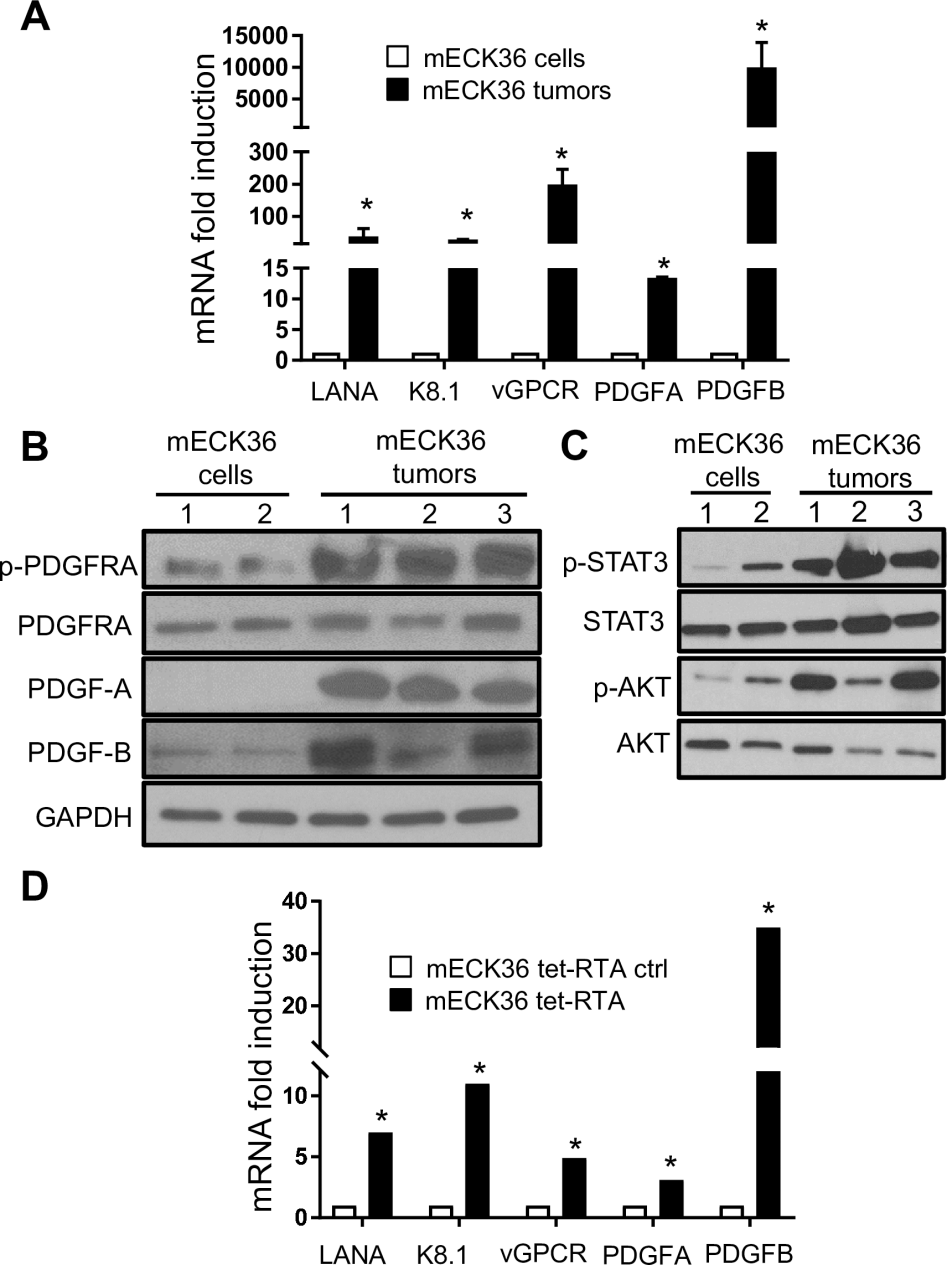
KSHV-mediated PDGF upregulation and PDGFRA activation in mECK36 cells and tumors. (**A**) Fold-changes in KSHV gene expression and PDGFRA ligands between mECK36 cells and mECK36 tumors determined by RT-qPCR in triplicate and are presented as means ± SD. *P < 0.05. (**B**) Total and phospho-PDGFRA together with its ligands PDGFA and PDGFB determined by immunoblotting in mECK36 cells (duplicate) and three mECK36 tumors from 3 different mice.(**C**) Total and phospho-STAT3 together with Total and phospho-AKT were determined by immunoblotting in mECK36 cells (duplicate) and three mECK36 tumors from 3 different mice.(**D**) Fold-changes in PDGFRA ligands and KSHV gene expression between doxyxcyclin induced and un-induced mECK36 cells stably transfected with a Tet-inducible RTA were measured by RT-qPCR after 24 hours of induction. Data were from three independent experiments carried out in triplicate and are presented as means ± SD. *P < 0.05.

We next searched for mechanisms of PDGFRA signaling activation by KSHV. Previously published comparative analyses of mECK36 cells and tumors have shown that in this mouse model KSHV tumorigenesis is *in vivo*-restricted, and occurs with concomitant upregulation of KSHV lytic genes and angiogenic ligands/receptors [21]. We found that, compared to mECK36 cells, mECK36 tumors displayed a robust upregulation of lytic genes RTA, vGPCR and K8.1, which correlated with a 15 fold PDGFA and 5000 fold PDGFB upregulation (Fig 3A). Importantly, western blot analyses showed that along with these augmented levels of lytic gene expression, PDGFA and PDGFB correlated with prominent PDGFRA phosphorylation in tumor samples (Fig 3B), suggesting that it could be a result of lytic induction occurring during *in vivo* tumorigenesis. Moreover; we found activation of downstream signaling cascades modulated by PDGFR including AKT and also STAT3, an oncogenic signaling cascade which we previously found is potently activated by PDGF in mECK36 tumors [35] (Fig 3C). To assess the capability of KSHV lytic induction to cause PDGF ligand upregulation, we used mECK36 cells stably transfected with tetracycline-inducible RTA, the KSHV replication transcriptional activator gene that induces the switch from latency to lytic replication. This system was recently employed to create the efficient iSLK KSHV production system [36]. As shown in Fig 3D, KSHV lytic induction, characterized by upregulation of the KSHV early lytic genes vGPCR and the late lytic gene K8.1, occurred concomitantly with a marked upregulation of PDGFA and PDGFB expression (Fig 3D). Taken together, these *in vivo* and *in vitro* results show the existence of a mechanism for ligand-mediated activation of PDGFRA signaling triggered by KSHV lytic gene expression.

### KSHV vGPCR can activate PDGFRA by upregulation of its ligands

**Fig 4:**
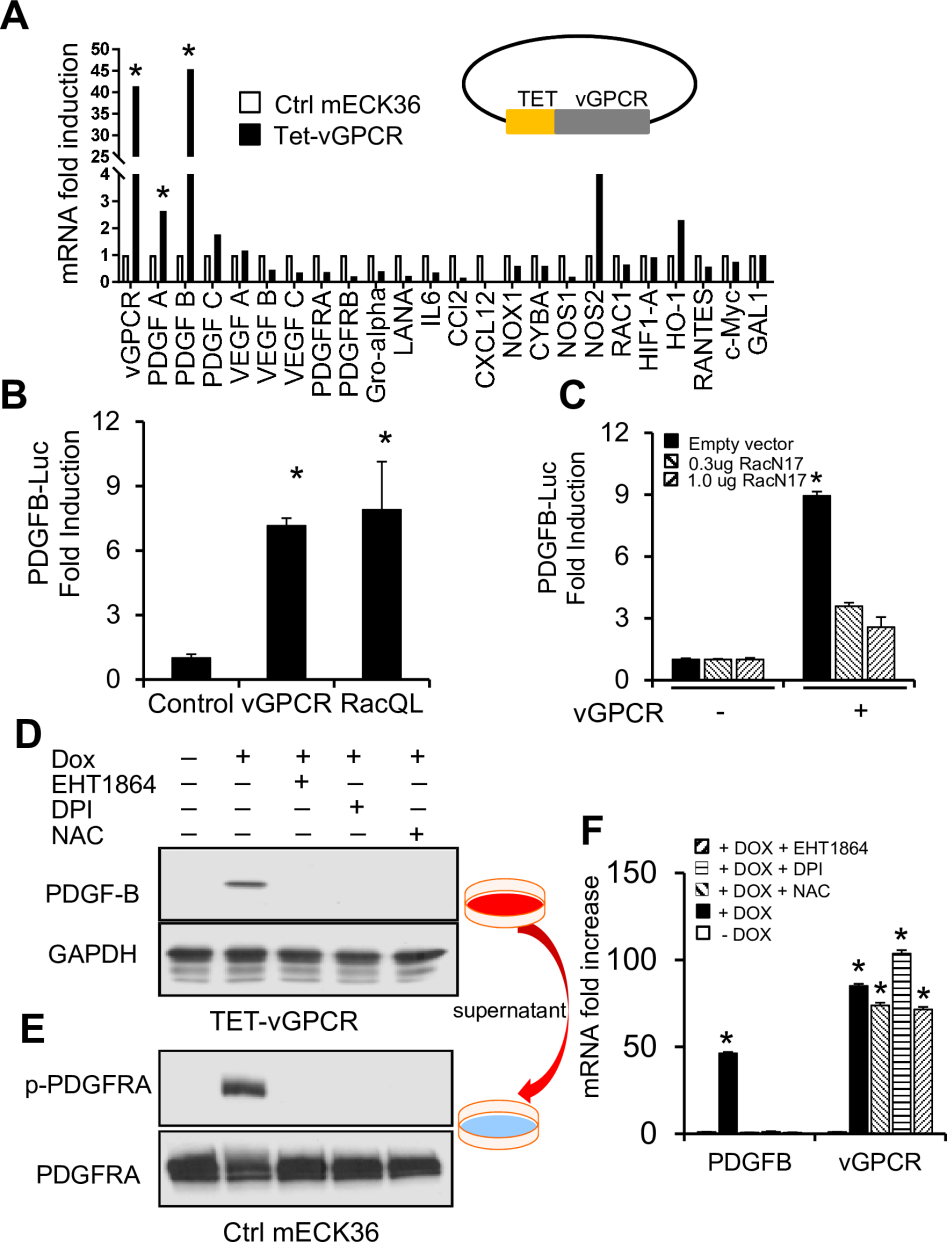
KSHV vGPCR can activate PDGFRA by upregulation of its ligands PDGFA/B in mECK36 cells. (**A**) mRNA levels determined by RT-qPCR of angiogenesis factors, cytokines and REDOX genes in Tetracycline-inducible vGPCR (TET-vGPCR) and control mECK36 stimulated with doxycycline for 24 hours. Data were from three independent experiments carried out in triplicate and are presented as means ± SD. *P < 0.05. (**B**) and (**C**) PDGFB-Luc promoter activity in 293T cells co-transfected with empty vector (control), vGPCR, and either a constitutively activated Rac1 (RacQL) construct (B) or a dominant negative Rac1 (RacN17) (C). Data were from three independent experiments carried out in triplicate and are presented as means ± SD. *P < 0.05. (**D**) Western blot analysis for PDGFB of TET-vGPCR mECK36 cells induced with doxycycline for 24 hrs in the presence of Rac1 inhibitor EHT1864, the NOX inhibitor DPI, or the ROS scavenger NAC. (**E**) Phosphorylated and total PDGFRA levels of mECK36 cells stimulated with conditioned media from TET-vGPCR mECK36 cells induced with DOX in the presence or the absence of Rac1, NOX and ROS inhibitors. (**F**) PDGFB mRNA and vGPCR levels of cells in (D) determined by RT-qPCR. Data were from three independent experiments carried out in triplicate and are presented as means ± SD. *P < 0.05.

Our results *in vitro* and *in vivo* indicate that KSHV lytic replication is associated with upregulation of PDGF ligands and PDGFRA activation. Among KSHV lytic genes implicated in KS oncogenesis, KSHV vGPCR was shown to activate angiogenic factors and inflammatory cytokine expression in several KS models [16, 19, 20, 37]. In fact, shRNA silencing experiments in our mECK36 system showed that vGPCR is critical for angiogenesis and KS-like tumorigenicity [21]. Therefore, we tested if vGPCR can induce the expression of PDGF ligands in KSHV-infected mECK36 cells *in vitro*. Long term cultures of mECK36 cells become tightly latent and express negligible levels of vGPCR; thus, to study the effects of vGPCR expression in the context of KSHV infection and to mimic the robust vGPCR upregulation observed in mECK36 tumors (Fig 3A), we used a tetracycline-inducible vGPCR (TET-vGPCR) mECK36 cell line. Using a RT-PCR panel that includes many angiogenesis-related genes, we found that upregulation of vGPCR increased the expression of few angiogenic genes. Yet, interestingly, among the most upregulated angiogenic genes were the PDGFRA ligands PDGFB and PDGFA (Fig 4A). PDGF mRNA upregulation was due to increased transcription, as shown by stimulation of a PDGFB-Luc construct (Fig 4B). We previously found that vGPCR signals via Rac1 activation of ROS production by NADPH oxidase (NOS) [35]. Since PDGFB is known to be a ROS-regulated gene [38], we used genetic tools to test if Rac1 was involved in vGPCR-upregulation of PDGFB transcription. For this, we used a dominant-negative form of Rac1 (RacN17), which blocked vGPCR stimulation of PDGFB transcription in a dose-dependent manner (Fig 4C). Further evidence of Rac1 involvement in PDGFB upregulation was that a constitutively active Rac1 mutant (RacQL) was also able to increase the PDGFB-promoter activity (Fig 4B). To determine if PDGF ligands secreted by KSHV-infected cells overexpressing vGPCR activate PDGFRA signaling, we analyzed by western blot and tested the effect of conditioned media from TET-vGPCR mECK36 cells in mECK36 cells. We found that only DOX-induced Tet-vGPCR mECK36 cells expressed the PDGFRA ligand PDGFB (Fig 4D). Consequently, we found that only their supernatant induced phosphorylation of the PDGFRA receptor in mECK36 cells (Fig 4E). This indicates that KSHV vGPCR could activate PDGFRA in an autocrine and/or paracrine manner through the secretion of PDGF.

We further established that the vGPCR-mediated upregulation of PDGF was driven by a Rac1-ROS-NADPH oxidase (NOX) axis by the use of inhibitory drugs. Fig 4D–4F shows that addition of the Rac1 inhibitor EHT1864, the NOX inhibitor diphenyleneiodonium chloride (DPI), or the ROS scavenger NAC blocked vGPCR-mediated induction of PDGFB expression in TET-vGPCR mECK36 cells without affecting vGPCR levels of expression. This indicates that vGPCR induction of PDGFB requires Rac1 and NOX mediated ROS production. Taken together these data point to vGPCR and its downstream oxidative signaling effectors as one of the potential KSHV-driven mechanism for activation of PDGFRA through its ligands.

### PDGFR activated ROS signaling stimulates angiogenesis, cell proliferation, latent gene expression and tumorigenesis

**Fig 5:**
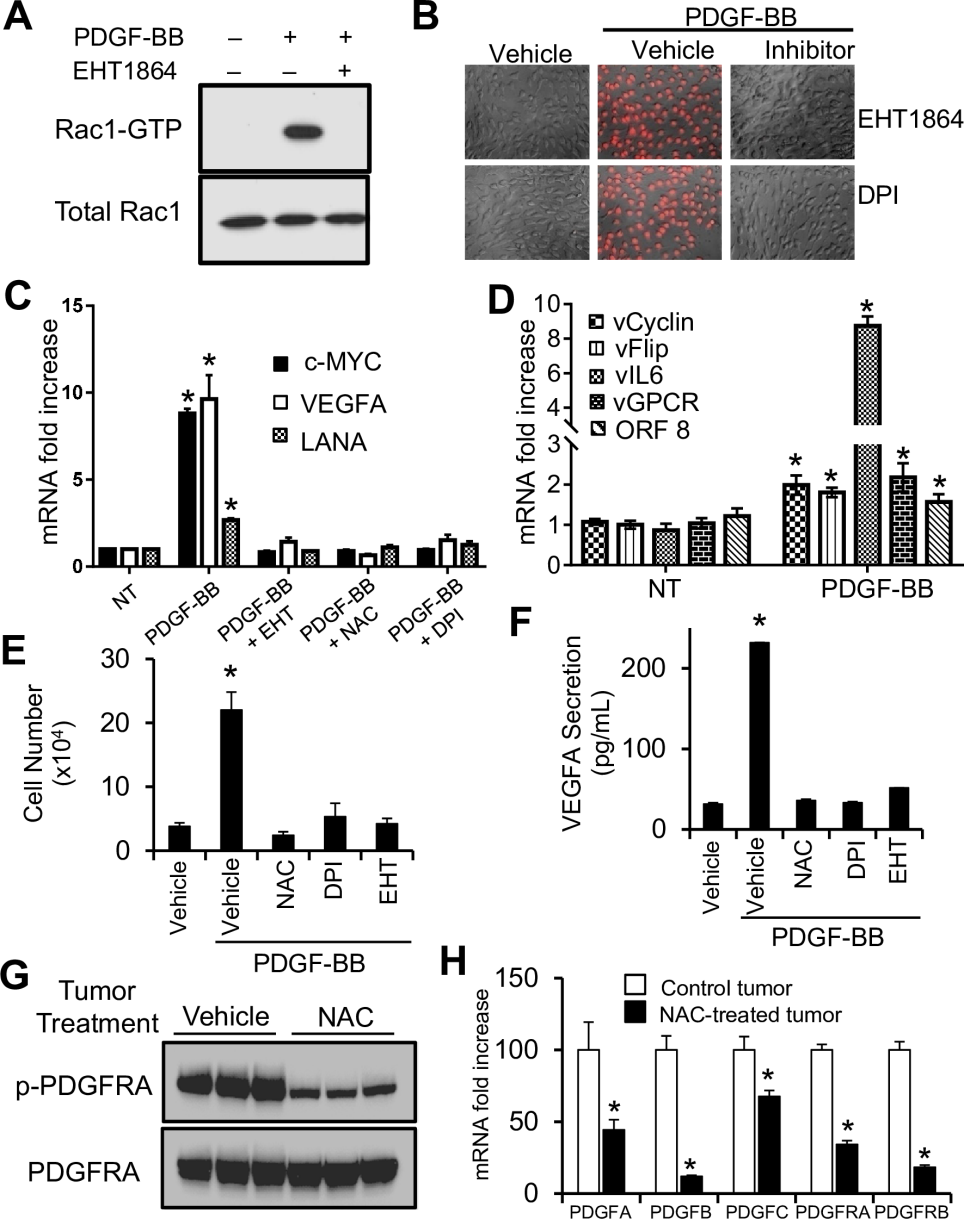
PDGFR signaling drives proliferation and angiogenesis in mECK36 cells and tumors via a Rac1-ROS-NOX dependent pathway. (**A**) Rac1 activation levels determined by a GTP-bound Rac1 pull-down assay/ Rac1 immunoblotting of mECK36 cells treated with PDGF-BB in the presence or in the absence of Rac1 inhibitor EHT1864. (**B**) ROS production (superoxide) of mECK36 cells stimulated with PDGFBB. Rac1 inhibitor (EHT1864) or NOX inhibitor (DPI) were added before PDGF stimulation (**C**) mRNA expression levels of c-Myc, VEGFA and KSHV LANA determined by RT-qPCR of mECK36 cells stimulated with PDGF-BB in the presence of absence of Rac1 inhibitor EHT1864, ROS scavenger NAC, or NOX inhibitor DPI. Data were from three independent experiments carried out in triplicate and are presented as means ± SD. *P < 0.05. (**D**) mRNA expression levels of KSHV v-Cyclin, v-FLIP, v-IL6, vGPCR and ORF8 determined by RT-qPCR of mECK36 cells stimulated with PDGF-BB. Data were from three independent experiments carried out in triplicate and are presented as means ± SD. *P < 0.05. (**E**) and (**F**) Proliferation (E) or VEGF secretion measured by ELISA (F) of mECK36 cells stimulated with PDGF-BB in the presence or absence of NAC, DPI or EHT1864. Data were from three independent experiments carried out in triplicate and are presented as means ± SD. *P < 0.05. (**G**) Phosphorylated and total PDGFR levels in NAC-treated and control mECK36 tumors were determined by immunoblotting. (**H**) mRNA levels of PDGFs and PDGFRs in NAC treated and control mECK36 tumors were determined by RT-qPCR. Data were from three tumors per treatment and are presented as means ± SD. *P < 0.05.

Our results of Figs 2 to 4 show that a plausible mechanism of PDGFRA activation by KSHV can be mediated through upregulation and secretion of its ligands. Therefore, to model the oncogenic effects of PDGFRA signaling we treated cultured mECK36 cells with PDGFBB and assessed oxidative signaling, proliferation and angiogenesis. We found that PDGFBB stimulation of KSHV-infected mECK36 cells led to Rac1 activation which was counteracted by the Rac1 inhibitor EHT1864 (Fig 5A). Both the Rac1 inhibitor, EHT1864, and the NOX inhibitor, DPI, blocked ROS production triggered by PDGF, further showing the involvement of Rac1 and NOX in PDGFR induction of ROS (Fig 5B). Molecular analysis of mECK36 cells treated with PDGF showed the upregulation of genes that control cell proliferation and angiogenesis, such as c-Myc, and the angiogenic and KS-growth factor vascular endothelial growth factor (VEGFA), as well as the KSHV LANA. Such transcripts were regulated in a Rac1-and ROS-dependent manner as treatment with EHT1864, NAC, and DPI blocked their induction by PDGFBB (Fig 5C). PDGFBB led to a general activation of KSHV gene expression which included v-cyclin, v-FLIP, v-IL6, vGPCR and ORF8 (Fig 5D). Importantly, PDGF stimulation induced proliferation of mECK36 cells, which was inhibited by NAC, DPI, and EHT1864 treatments (Fig 5E). ELISA analysis of PDGFBB-stimulated mECK36 cells revealed increased levels of VEGF secretion, which was blocked by NAC, DPI, and EHT1864, as well (Fig 5F). Taken together, these results point to a role for PDGF stimulation of PDGFRA oxidative signaling on the activation of angiogenesis, cell proliferation, and viral gene expression in KSHV oncogenesis. In our prior attempt to rationally design therapies for KS, we showed that by targeting oxidative signaling by NAC, we were able to inhibit KSHV oncogenesis in the mECK36 model [35]. We showed that NAC treatment does not affect the expression of key KSHV genes [35]; nevertheless, results of Figs 4 and 5 predicts that one of the possible mechanisms whereby NAC inhibits KSHV tumorigenesis is by targeting the mechanism of PDGFRA activation and its downstream signaling, which are both mediated by ROS[39]. To test this possibility, we carried out an immunoblotting analysis of the PDGFR in the shrinking tumors treated with NAC, which showed a reduction in phosphorylation levels when compared to control-treated tumors (Fig 5G) Interestingly, NAC-treated tumors displayed significantly reduced levels of both PDGF ligands and receptors (Fig 5H) indicating that NAC anti-tumorigenicity could be explained on the basis of its ability to target PDGFRA expression as well as its activation.

### Targeting PDGFRA signaling with RTK inhibitory drugs blocks KSHV oncogenesis

**Fig 6:**
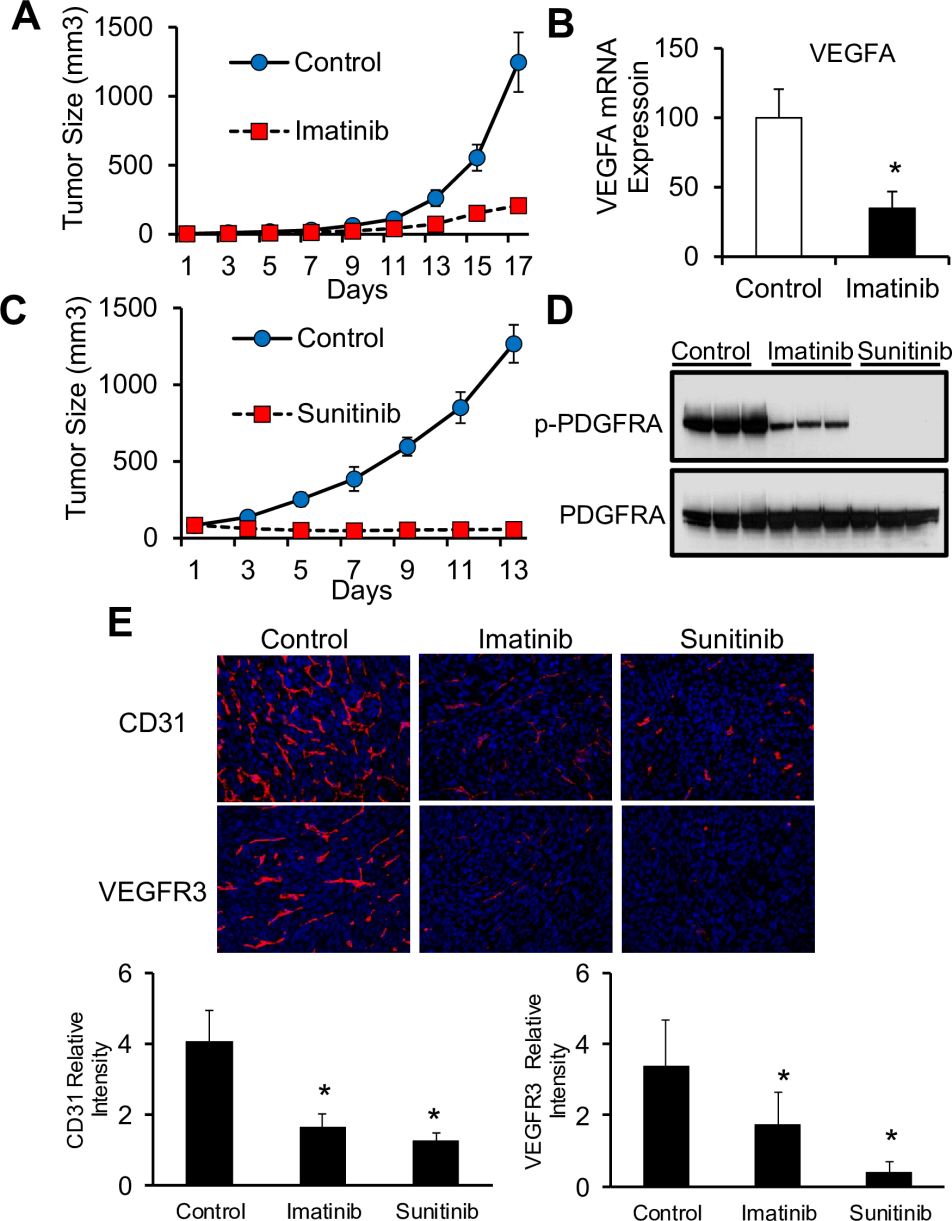
Multi tyrosine kinase inhibitors that can target PDGFRA block KSHV-mediated tumorigenesis. (**A**) Tumor growth curve from mice with established subcutaneous mECK36 tumors treated with vehicle (PBS) or Imatinib (150 mg/Kg twice daily) by oral administration. Data indicate mean tumor size ± SD (n = 10). (**B**) mRNA levels of VEGFA in Imatinib treated and control mECK36 tumors determined by RT-qPCR. (**C**) Tumor growth curve from mice with established subcutaneous mECK36 tumors treated with vehicle (PBS) or Sunitinib (80 mg/Kg/day) by oral administration. Data indicate mean tumor size ± SD (n = 10). (**D**) Phosphorylated and total PDGFR levels from Imatinib-treated, Sunitinib-treated and control mECK36 tumors determined by immunoblotting. (**E**) IFA of frozen sections of Imatinib-treated, Sunitinib-treated and control tumors stained with pan-endothelial marker CD31 (red) or lymphatic microvessel marker VEGFR3 (red). Nuclei were counterstained with DAPI (blue). Bar graphs show the relative intensity signals of CD31 and VEGFR3 from 10 different fields. *P < 0.05.

The prominent activation of PDGFRA in KSHV-infected tumors and the data shown in Fig 5 reinforces the concept supported by the promising results of the Imatinib trials [33, 34], indicating that the PDGFR pathway is an attractive target for drug therapy in KS. To further validate our mECK36 animal model as a potential preclinical model to test PDFGRA-targeted therapies to KS, we tested the efficacy of Imatinib Mesylate (Gleevec), a FDA-approved Bcr-Abl, c-kit, and PDGFR inhibitor that showed tumor responses in Phase I and II AIDS-KS clinical trials [33, 34], in the mECK36 tumorigenicity model. We found that, as expected from the similarities between mECK36 tumors and KS [21], oral administration of Imatinib to mice bearing mECK36 tumors was anti-tumorigenic (Fig 6A). Furthermore, in agreement with our results of Fig 5 showing that PDGF signaling upregulates VEGF in KSHV-infected cells, we found decreased VEGF mRNA levels in Imatinib-treated tumors and cells (Figs 6B and S1). These findings point to Imatinib as both an anti-tumorigenic and an anti-angiogenic drug in the context of KSHV oncogenesis. Yet, we found that even at the highest dose employed, Imatinib only partially inhibited PDGFRA phosphorylation and did not totally prevent mECK36 tumor growth (Figs 6D and 6A and S1). To increase the tumoricidal effects we tested the poly RTK inhibitor Sunitinib [40], a drug approved for renal cell carcinoma and tested in Phase I clinical trials for tumors in the context of HIV/AIDS [41]. Sunitinib has a higher inhibitory activity to PDGF receptor and more anti-angiogenic activity since it is able to target VEGF receptors (S1 Table). We first compared, *in vitro*, the potency of the drugs and their ability to inhibit PDGFR-induced cell proliferation and VEGF secretion. As shown in supplementary Fig 1 Sunitinib displayed a much more potent anti-proliferative and anti-VEGF effect that correlated with its ability to completely block PDGFRA phosphorylation. As shown in Fig 6C, Sunitinib treatment of mECK36 tumors showed a stronger anti-tumor effect than Imatinib, leading to a complete inhibition of tumor growth, which occurred with complete blockage of PDGFRA phosphorylation (Fig 6D). Since Sunitinib can inhibit the VEGFRs and target neovascularization, we analyzed Imatinib- and Sunitinib-treated tumors for the pan-endothelial (CD31) and lymphatic-endothelial (FLT4/ VEGF-R3) markers. We found that CD31 and VEGFR3-expressing microvessels were significantly decreased by all treatments (Fig 6E), and that Sunitnib led to a stronger neo-vessel growth inhibition than Imatinib. Taken together our results show that Sunitinib’s stronger PGFRA inhibition combined with its anti-angiogenic activity leads to a more efficacious inhibition of mECK36 tumor growth.

### Maintenance of tumorigenesis in KSHV-ve mouse KS through PDGFRA activating mutations

**Fig 7:**
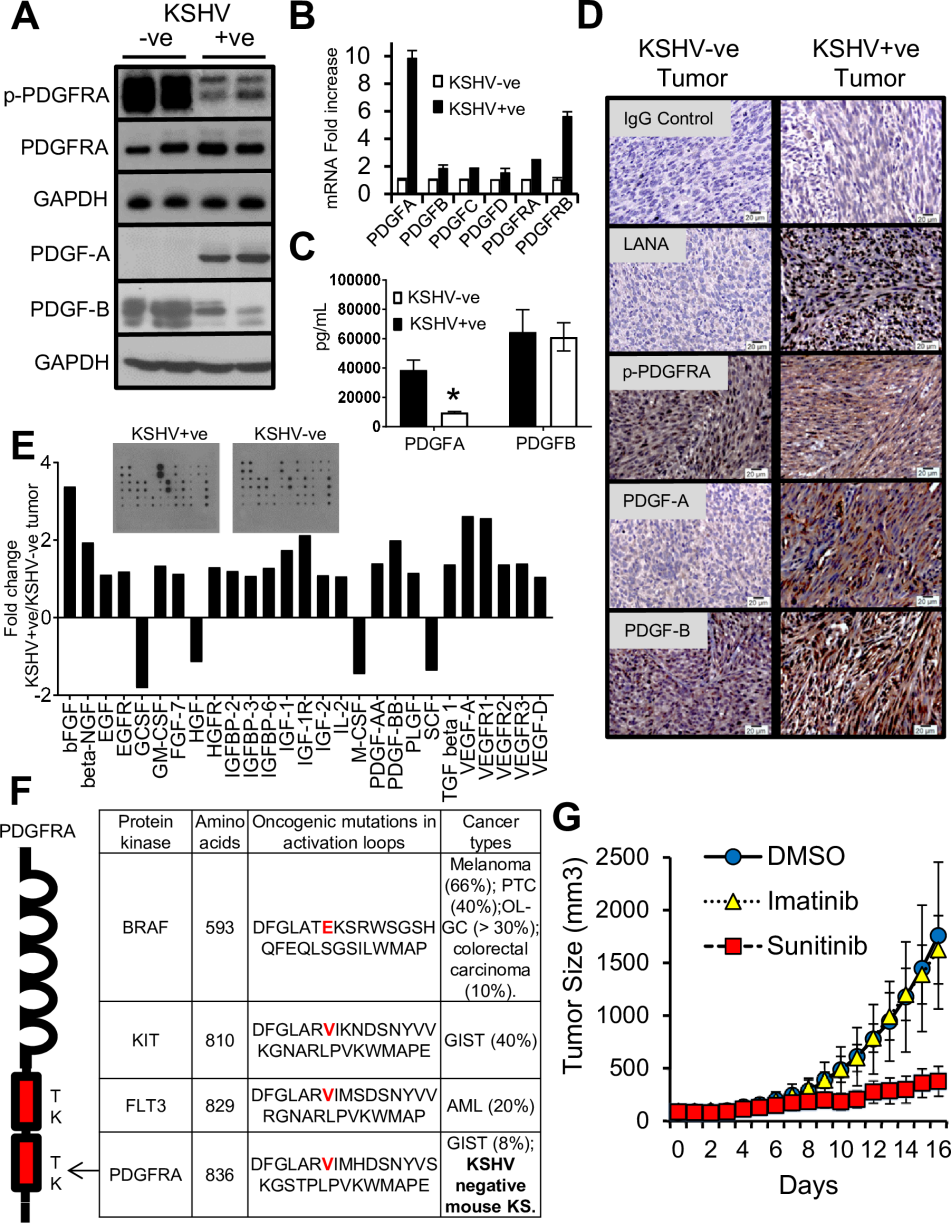
Maintenance of tumorigenesis in KSHV-negative mECK36 tumors through PDGFRA activating mutations. (**A**) Phosphorylated PDGFRA, total PDGFRA, PDGFA and PDGFB levels from KSHV+ve mECK36 and KSHV-ve mECK36 tumors determined by immunoblotting. (**B**) mRNA levels of PDGFs and PDGFRs in KSHV+ve mECK36 and KSHV-ve mECK36 tumors determined by RT-qPCR. Data are from three tumors carried out in triplicate and are presented as means ± SD. *P < 0.05. (**C**) ELISA of Platelet-Derived Growth Factor AA (PDGF-AA) and Platelet-derived growth factor subunit BB (PDGF-BB) in KSHV+ve mECK36 and KSHV-ve mECK36 tumor tissues. Data are from three tumors and are presented as means ± SD. *P < 0.05. (**D**) Immunohistochemistry staining of KSHV+ve mECK36 and KSHV-ve mECK36 tumor tissues using antibodies against PDGFA, PDGFB, LANA, and phospho-PDGFRA. (**E**) Mouse Growth Factor Antibody Array used to detect 30 Mouse Growth Factors in KSHV+ve and KSHV-ve tumors. Data is presented as fold change expression between KSHV+ve mECK36 and KSHV-ve mECK36 tumor tissue. (**F**) Sequence alignment of the hot spot region for oncogenic mutations in the activation domain of TKs. The D842V mutation in PDGFRA was only found in the cDNA of tumorigenic KSHV-negative mECK36 cells. (**G**) Tumor growth curve from mice with established subcutaneous KSHV-negative mECK36 tumors treated with Imatinib (150 mg/Kg twice daily) or Sunitinib (80 mg/Kg/day) by oral administration. Data indicate mean tumor size ± SD (n = 10).

mECK36 cells represent a model of KSHV-dependent tumorigenesis. When mECK36 cells lose the KSHV episome *in vitro* by withdrawal of antibiotic selection, they completely lose tumorigenicity [21]. However, explanted mECK36 tumor cells that are forced to lose the KSHV episome *in vitro* are tumorigenic (KSHV-ve mECK36) [35]. This is likely due to host genetic alterations accumulated during *in vivo* tumor growth that can compensate for KSHV tumorigenicity after loss of the KSHV episome. We found that KSHV-ve mECK36 tumors were histopathologically and transcriptionally close to KSHV+ve mECK36 tumors, however they produced tumors that were resistant to NAC treatment [35]. Since the PDGFRA activation axis appears to be essential in KSHV tumorigenesis, we compared the molecular and activation status of the PDGF-PDGFR axes in tumors induced by KSHV+ve and KSHV-ve cells. Although both tumors displayed PDGFRA activation (Fig 7A and 7D), in the case of KSHV-negative tumors PDGFRA activation was extremely pronounced and occurred in the context of a much lowered expression and production of the PDGFRA specific ligand PDGFA as shown by western blot and IHC (Fig 7A and 7D). These results were also confirm by an ELISA analysis to quantify the PDGF content of tumors (Fig 7C). To determine the impact of KSHV infection in the levels of cytokines and angiogenic growth factors and receptors we employed a growth factor array to compare KSHV+ve mECK36 with KSHV-ve mECK36 tumors. We found a global upregulation of growth factors and their receptors in KSHV+ve mECK36 tumors (Fig 7E), including upregulation of PDGFA and PDGFB expression, bFGF, IGF, VEGFs and its receptors 1,2 and 3. Yet; in spite of the upregulated levels of this paracrine and angiogenic mediators and its receptors, they failed to displayed the very robust levels of receptor activation shown in Fig 1 for PDGFRA and PDGFRB, further reinforcing the idea of the predominance of PDGFR oncogenic signaling in KSHV-infected KS-like tumors.

A likely explanations for the strong PDGFR phosphorylation in KSHV-negative tumors could be the increased PDGFB ligand expression as well as the occurrence of oncogenic mutations that convey constitutive signaling, like those found in other PDGFRA-driven sarcomas such as GIST [27, 28]. We therefore sequenced the full-length cDNA of PDGFRA and PDGFRB in KSHV+ve and KSHV-ve mECK36 cells and their corresponding tumors. PDGFRB was wild-type in all samples. PDGFRA was wild-type in KSHV+ve tumors and cells. In contrast, KSHV-ve tumors and cells had a heterozygous D842V mutation in the tyrosine kinase (TK) domain of the PDGF receptor alpha (Fig 7F). Interestingly, D842V is the most common activating mutation of PDGFRA found in GIST [27], which occurs in the PDGFRA activation domain that is a hot-spot for oncogenic mutations in many RTKs (Fig 7F), and was found to confer Imatinib resistance in GIST [27, 42]. As shown in Fig 7G, KSHV-ve tumors were indeed resistant to Imatinib. This data further support the idea that PDGFRA is an oncogenic driver in KSHV tumors. D842V mutations are known to also confer resistance to Sunitinib in GIST [43]. We speculated that since in the context of the mECK36 model, Sunitinib was also potently anti-angiogenic, this activity might be sufficient to target PDGFRA D842V-driven tumors. As shown in Fig 7E, D842V mutant KSHV-ve mECK36 tumors that were resistant to Imatinib showed sensitivity to Sunitinib, suggesting that concomitant multi-kinase inhibition, including angiogenic receptors, might also be able to overcome drug resistance.

### PDGFR signaling plays a necessary role in KSHV oncogenesis

**Fig 8:**
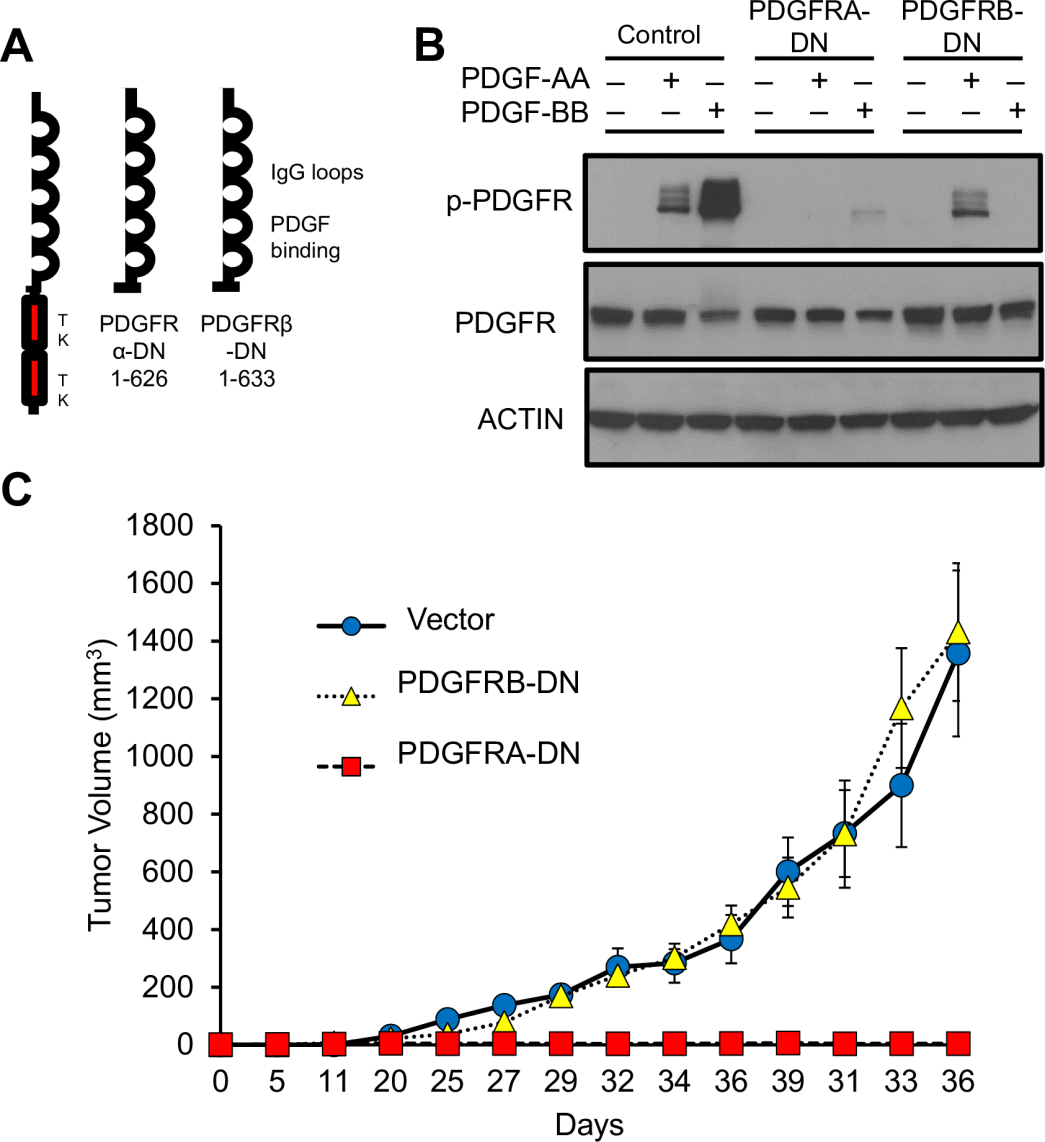
Tyrosine-kinase truncated dominant-negative mutants of PDGFRA block KSHV tumorigenesis in mice. (**A**) Structure of the truncated dominant-negative forms of PDGFRA and PDGFRB. (**B**) Phosphorylated PDGFR and total PDGFR determined by immunoblotting in mECK36 cells transfected with dominant-negative forms of PDGFRA, PDGFRB, or empty vector control stimulated with either PDGF-AA (80 ng/mL) or PDGF-BB (20ng/ml) for 10 min. (**C**) Tumor growth curve from mice following subcutaneous injection of mECK36 cells transfected with dominant-negative forms of PDGFRA, PDGFRB, or empty vector control. Data indicate mean tumor size ± SD (n = 10).

The experiments with multi-kinase PDGFR-inhibitory drugs Imatinib and Sunitinib, show that inhibition of PDGFR signaling correlated with an anti-tumor effect. To specifically establish a necessary role for PDGFR signaling in KSHV tumorigenesis, we devised a dominant negative (DN) strategy based on the classic approach published by Rusty Williams and collaborators [44, 45]. mECK36 cells were transfected with an EF1-driven, puromycin-resistant expression vector encoding a TK domain-truncated DN form of PDGFRA and PDGFRB or an insert vector control (Fig 8A). To test the biological ability of these DNs to affect PDGFR signaling and tumor growth, we assessed the ability of these DN mutants to block PDGF stimulation of PDGFR phosphorylation. We found that, as described in the original publication, PDGFRB DN only blocked PDGFB stimulation but failed to block PDGFA stimulation (Fig 8B) [44, 45]. In contrast, the PDGFRA DN strongly inhibited both PDGFA and PDGFB signaling (Fig 8B). After a round of puromycin selection and EGFP cell sorting, DN or vector control transfected mECK36 cells, which displayed similar viability and ability to grow *in vitro*, were injected into nude mice. We found that both the control vector and the PDGFRB DN constructs displayed similar tumorigenicity as their parental mECK36 cells, giving rise to KSHV+ve tumors at day 15 (Fig 8C). In stark contrast, cells transfected with the PDGFRA DN did not grow tumors (Fig 8C). These inhibition results, in combination with the kinome results of Fig 1A, conclusively show a critical involvement of PDGFRs in KSHV-induced tumorigenesis, further supporting its candidacy as a KS oncogenic driver. This indicates that approaches that fully and stably inhibit PDGFR-signaling could be successful therapeutic strategies in KS.

### PDGFRA is expressed and phosphorylated in KSHV-infected spindle cells of the majority of AIDS-KS biopsies

**Fig 9:**
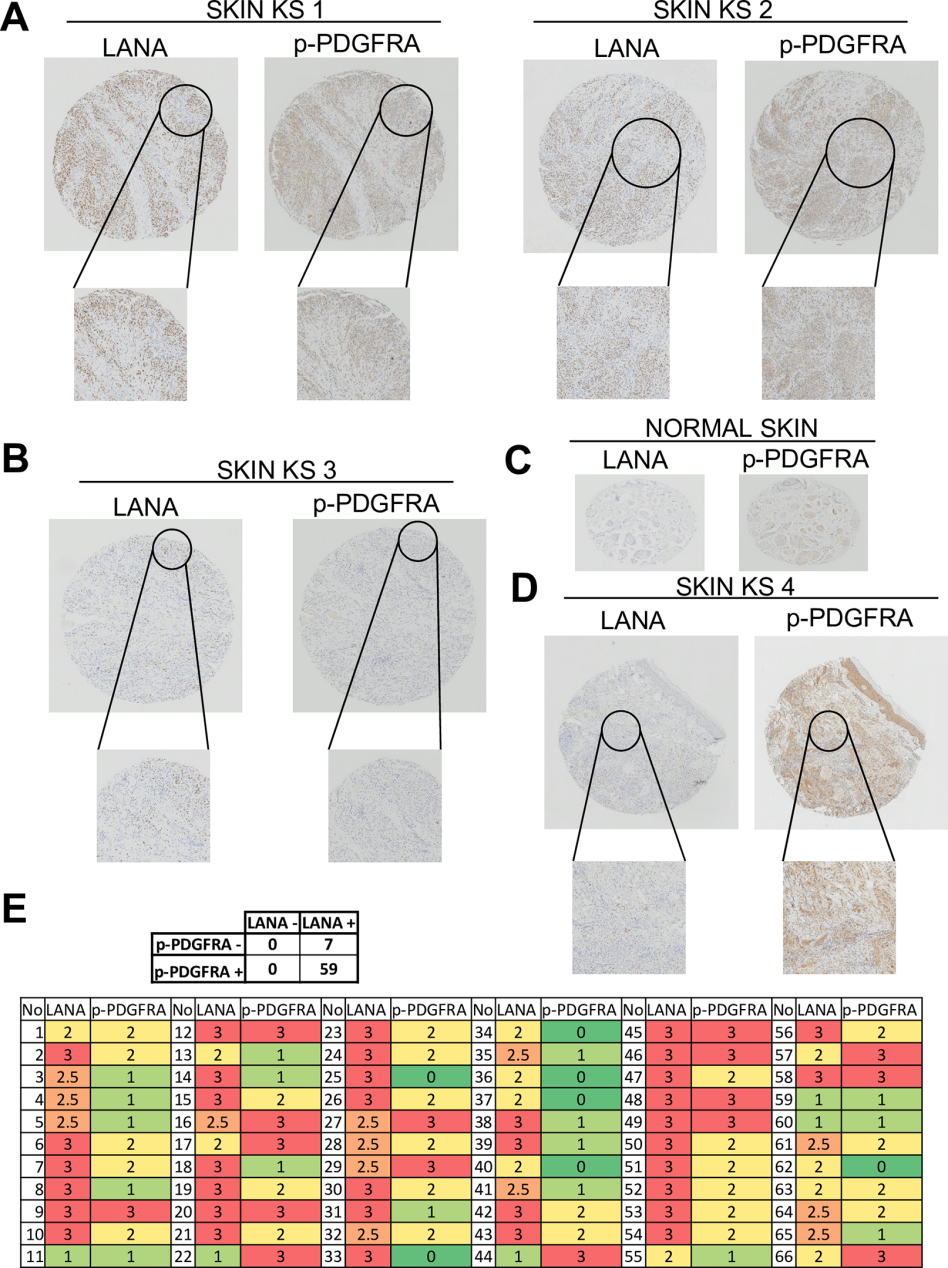
PDGDRA phosphorylation is consistently found in spindle-cells of AIDS-Kaposi’s sarcoma lesions and localizes to areas of KSHV infection. (**A**) Staining AIDS-KS biopsies from a ACSR tissue microarray (TMA) showing that phospho-PDGFRA localizes to areas of LANA staining in two characteristic samples of the 59 out of 66 skin KS tumors which were strongly phospho-PDGFRA+ve/ LANA+ve. (**B**) Example of one of the 7 phospho-PDGFRA-ve (LANA+ve) AIDS-KS tumors of the TMA. (**C**) Normal control tissue from the TMA (skin). (**D**) Example of a KS tumor with strong phospho-PDGFRA staining and a low percentage of LANA+ve cells. (**E**) LANA and phospho-PDGFRA were scored from 0 to 3 depending on the signal strength of the antibody staining (bottom table) and total number of PDGFRA+ve/LANA+ve (59) and PDGFRA-ve/LANA+ve (7) biopsies are shown over the 66 skin AIDS-KS biopsies analyzed (top table).

Our results in the mECK36 model provide strong evidence for the critical role that KSHV-induced activation of PDGFRA signaling has in KSHV tumorigenesis. These results suggest that such a signaling pathway operating in KS lesions could be critical in tumorigenesis and as a therapeutic target. To determine the prevalence of PDGFRA activation in AIDS-KS we stained for KSHV LANA and phospho-PDGFRA in skin KS biopsies and controls contained in a tissue microarray developed by the AIDS Cancer Specimen Repository (ACSR). We consistently found that in the majority of the cases, areas that stained strongly for phosphorylated PDGFRA also stained for nuclear KSHV-LANA (exemplified in Fig 9A and quantified in table of Fig 9E). This strong co-localization occurs in 59 out of 66 KS biopsies tested in this array. Only 7 of the analyzed samples exhibited LANA+ve and phospho-PDGFRA-ve (Fig 9B), while normal skin exhibited negative LANA staining and weak phospho-PDGFRA staining in some areas (Fig 9C). Among other KS skin biopsies obtained through our Dermatopathology service, we were able to find a few biopsies in which only a small percentage of phospho-PDGFRA+ve cells were LANA positive (Fig 9D). This can be explained on the basis of the existence of paracrine PDGFRA activation by KSHV infected cells. Taken together, our results indicate that prominent PDGFRA phosphorylation occurs in KSHV-infected spindle cells of most AIDS-KS tumors; suggesting that most AIDS-KS patients could be amenable for therapeutic interventions targeting this oncogenic mechanism.

## Discussion

**Fig 10:**
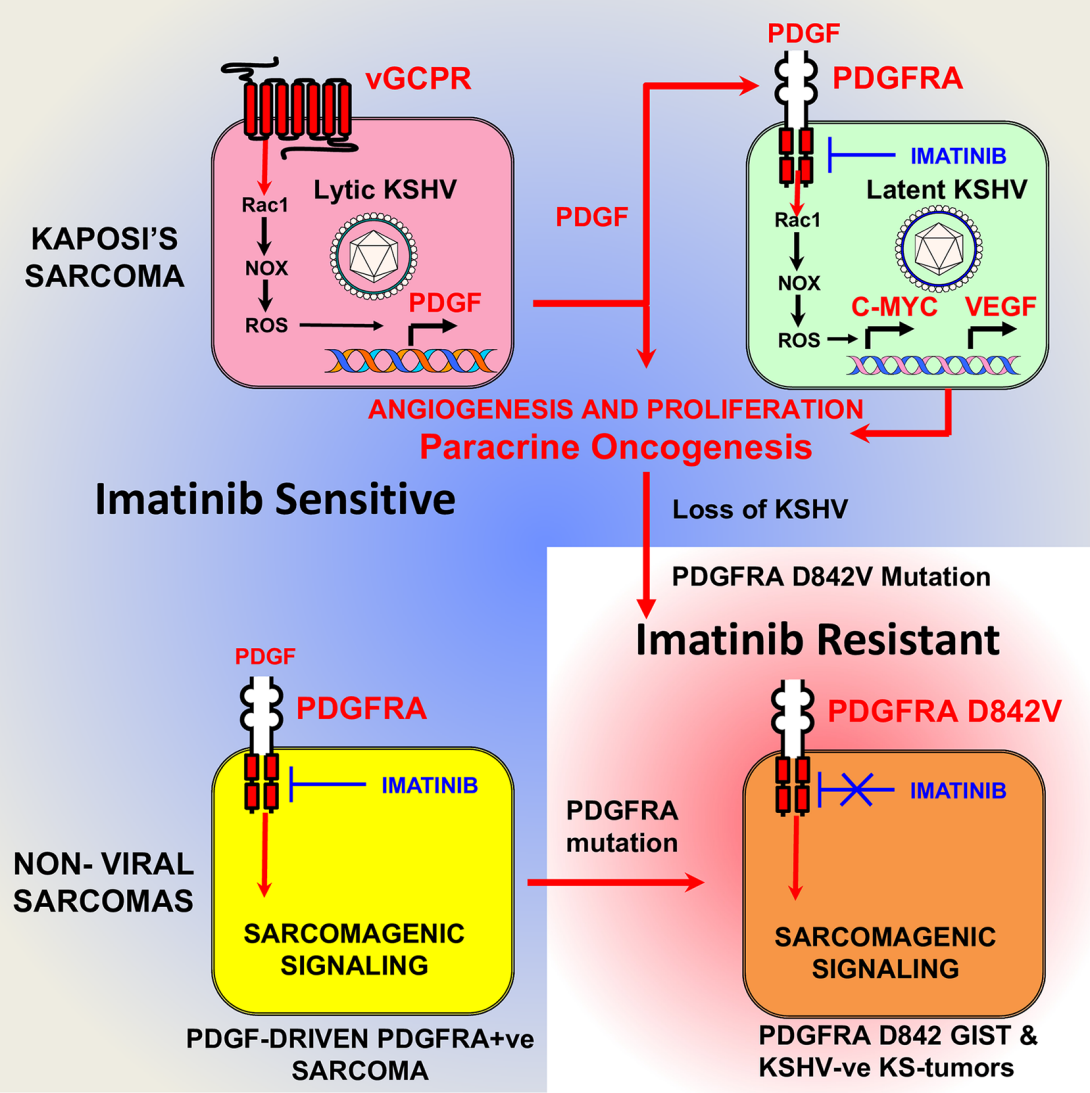
KSHV-dependent and independent mechanisms of PDGFRA-driven sarcomagenesis. The upper panel describes one possible PDGFRA activation mechanism supported by data from this paper: lytically or abortive-lytically infected KS cells expressing vGPCR can drive sarcomagenesis by Rac1-NOX-ROS mediated upregulation of PDGF leading to PDGFRA activation in latently infected cells that promotes proliferation and VEGF angiogenesis (paracrine oncogenesis). This mechanism would be sensitive to Imatinib, which inhibits PDGFRA oncogenic signaling. The lower panels show in the left panel; the scenario for non-viral sarcomas driven by ligand-mediated activation of PDGFRA (soft tissue sarcomas) which are sensitive to Imatinib and to anti-PDGFRA antibody therapy [56]. The right panel shows the scenario for our KSHV-ve KS-like sarcomas driven by the PDGFRA D842V mutations—the most frequent PDGFRA mutation in GIST—which make these tumors Imatinib-resistant.

Using kinase proteomic arrays to rank host-signaling pathways activated in a mouse model of KSHV tumorigenesis, we found that activated PDGF receptor-alpha (PDGFRA) is a predominant oncogenic RTK in KS. We showed that KSHV can activate sarcomagenic PDGFRA signaling through upregulation of PDGFs by KSHV lytic genes including vGPCR, we show that blocking PDGFRA signaling is anti-tumorigenic and we show that PDGFRA is prominently phosphorylated in KS (Fig 10).

Further refining of previous analyses that have highlighted many paracrine and autocrine stimulatory pathways [3], we found in this study that PDGFRA activation accounted for the dominant share of RTK activation of mouse-KS tumors, strongly pointing to PDGFRA as a critical oncogenic driver. Nevertheless, it is relevant to mention that many RTKs, including PDGFRB, VEGFRs and c-kit (Fig 1), were also phosphorylated, yet to a lesser extent, and therefore, as suggested by the results of Fig 6, could also play an important role in KS tumorigenesis and angiogenesis.

The existence of a KSHV-induced, ligand-mediated mechanism for PDGFRA activation is supported by immunohistochemistry staining of mECK36 and AIDS-KS lesions showing that the areas staining for both KSHV-LANA and phospho-PDGFRA correspond with the detection of PDGFRA ligands PDGFA and PDGFB. The robust activation of PDGFRA signaling observed in mECK36 tumors compared to mECK36 cells *in vitro*, and in AIDS-KS lesions may be explained by the increased lytic gene expression found *in vivo* [21] also reported for a portion of KS lesions [46]. These mechanisms could be prominent during KSHV tumorigenesis acting in a paracrine manner from either lytically or abortive-lytically KSHV-infected cells that express PDGF upregulating oncogenes such as vGPCR [47] (Fig 10). PDGFRA activation could be further enhanced through interactions operating *in vivo*, such as KSHV-induced Notch activation, as well as other mechanisms that have been shown to upregulate PDGFR signaling, such as the E3 ubiquitin ligase ORF K5 [48] [3, 49]. Current experimental evidence cannot sort out if PDGFs are exclusively secreted by lytically infected cells, which are profuse in mECK36 tumors but are less prominent in established KS lesions where the majority of spindle cells are latently infected.

Since vGPCR is a potent inducer of KS growth factors, we used a Tet-inducible vGPCR construct to mimic the vGPCR-overexpression status of tumor forming mECK36 that display a 160 fold up-regulation of vGPCR mRNA levels. We found that vGPCR upregulates PDGF in mECK36 via a Rac1-NOX-ROS oxidative stress axis (Fig 10). In contrast to previous single gene and knock-out studies [16, 19, 21, 22], vGPCR overexpression did not lead to strong upregulation of VEGF mRNA in mECK36 cells. However, our results point to the enhancement of VEGF production downstream of PDGFRA signaling (see Fig 5) as a contributor to angiogenicity (Fig 10). We could not use siRNA vGPCR silencing to overcome the limitations of the vGPCR overexpression studies, because it is expressed at negligible levels in our current long term mECK36 cultures which are predominantly latent. To establish a direct link between vGPCR and ligand mediated PDGFRA activation we carried out experiments swapping Bac36 for Bac16 KSHV vGPCR-null mutants or its revertant. Paradoxically, long term cultures of vGPCR-null mECK16 upregulated PDGFs and PDGFRA signaling showing that, as suggested by our results; PDGFRA is a critical survival pathway that should be compensated even in the absence of vGPCR signaling via a mechanism—whose elucidation lies outside the scope of the present paper—that could be critical in maintenance of human KS lesions which are mostly latently infected and not expressing vGPCR.

The involvement of the Rac1-NOX-ROS pathway in vGPCR upregulation of PDGFB and in PDGFRA downstream signaling supports the role of oxidative stress in KSHV oncogenesis [35, 50]. This is further corroborated by NAC inhibition of mECK36 tumorigenesis, which we now show correlates with decreased levels of PDGF ligands, receptors and PDGFRA phosphorylation (Fig 5G and 5H). Moreover, in the absence of ROS, due to NAC neutralization, phosphatases are fully active and can effectively counteract the activity of RTK such as PDGFRA, which are known to require ROS for effective downstream signaling [39]. In previous studies we found that KSHV-ve mECK36 tumors were insensitive to NAC treatment and we found that NAC resistance correlated with upregulation of members of the Rac1-NOX signaling pathway [35]. Our current results show that the observed up-regulation of oxidative stress and NAC resistance, might be because KSHV-ve mECK36 tumors bears a PDGFRA activating D842V mutation (Fig 7), that is no longer dependent on the upregulation of the PDGF-PDGFRA loop targeted by NAC (Fig 4). On the other hand, in KSHV+ve mECK36 tumors PDGFRA activation appears to be driven by a KSHV-mediated ROS-dependent upregulation and activation of PDGF. Taken together, our results point to ROS signaling as critical for PDGFRA-driven oncogenesis, supporting the use of anti-oxidants as anti-KS agents (Fig 10).

In accordance with the promising results of the Imatinib AIDS-KS trials [33, 34], pharmacological intervention of PDGFR-signaling in mECK36 KS-like tumors, indicate that it is critical for KSHV-induced tumorigenesis. We found that Sunitinib displayed more potent anti-tumor effects in mECK36 tumors than Imatinib, which correlated with the extent of inhibition of PDGFRA phosphorylation achieved by the drugs (Fig 6). While Sunitinib is more active against PDGFR than Imatinib, the latter is more selective for PDGFR when compared to VEGF receptors (S1 Table) (Shurer S., Personal Commun.). Thus, it is unlikely that Imatinib inhibits angiogenesis by targeting VEGF receptors. The anti-angiogenic response obtained with Imatinib is likely due to its ability to decrease VEGF levels in cells and tumors (see Figs 6 and S1), by targeting PDGF ability to upregulate VEGF (Figs 5 and S1), and with the described pro-angiogenic activities of the PDGF family [51]. In the case of Sunitinib, it also has to do with the known strong anti-VEGFR inhibitory activities of the drug (S1 Table) [52]. Anti-tumor effects of Imatinib due to inhibition of c-Kit cannot be ruled out, since c-kit has been implicated in KSHV endothelial spindle cell transformation [53]. However, as our kinome proteomic study and western blots of Fig 1 show, there is negligible activation of c-kit in our KS model.

Our results point to a mechanism for PDGFRA activation in mECK36 tumors mediated by KSHV genes. We found that mECK36 explanted from tumors that have lost KSHV, induced tumorigenesis with very strong PDGFRA activation and low levels of PDGF-AA expression and secretion (Fig 7). We found that in KSHV-negative mECK36 tumorigenic cells PDGFRA display the activating mutation D842V, the most common PDGFRA mutation in GIST, which confers constitutive RTK activity and resistance to Imatinib [27, 42]. The occurrence of the D842V mutation among the host oncogenic alterations that compensate for KSHV-loss, and the fact that it can override Imatinib inhibition of the KSHV-ve tumor growth, strongly supports the oncogenic driver role of PDGFRA signaling in these tumors (Fig 10).

We resorted to genetic PDGFRA-signaling blockade using a tyrosine kinase truncated dominant negative mutant as a specific approach to suppress PDGFR signaling [44, 45]. The fact that the DN PDGFRA totally impeded mECK36 tumorigenesis validates PDGFRA as an oncogenic driver and it shows that approaches that fully and stably inhibit PDGFR-signaling could be successful therapeutic strategies in KS. PDGFRA is also an oncogenic driver in many non-viral sarcomas [27, 28]. Importantly, viral oncogenesis generally occurs with activation of the same oncogenic pathways that are deregulated in non-viral cancers of the same cellular lineage [12]. PDGF-driven activation of PDGFRA in synovial sarcomas [54] and activating mutations of PDGFRA in GIST [27] parallel the activation of PDGFRA in KSHV-positive and KSHV-negative mouse-KS, respectively. The fact that PDGFRA also drives non-viral sarcomas highlights the importance of KSHV-dependent activation of PDGFRA in KS. Furthermore, our findings provide a molecular basis to the histopathological classification of KS as true sarcomas (Fig 10).

The clinical relevance of our findings to KS is underscored by the analysis of AIDS-KS tumor biopsies that showed co-distribution of phosphorylated PDGFRA to areas infected with KSHV. Analysis of an AIDS-KS TMA showed strong phospho-PDGFRA staining in areas of KSHV-infected LANA+ve spindle cells in ninety percent of the biopsies (Fig 9), indicating that activated PDGFRA is consistently found in most skin AIDS-KS lesions. Sequencing of PDGFR and c-kit exons did not reveal activating mutations in the KS lesions of the patients treated with Imatinib (28), further suggesting that a KSHV-driven, PDGF-mediated mechanism maybe responsible for activation of PDGFRA in KS. In the last Phase II trial, more than 50% of patients benefited from Imatinib treatment [34]. This indicates that inhibition of PDGFR signaling has therapeutic benefit in AIDS-KS and further strengthens the idea that PDGFRA is an oncogenic driver in KS. Moreover, the results obtained in our models with Sunitinib and with dominant negative PDGFRA suggest that therapeutics approaches leading to complete inhibition of PDGFRA signaling can be highly efficacious in KS treatment.

The finding that a mutation occurring in KSHV-negative mouse-KS, which is known to confer Imatinib resistance [27, 42], suggests that such mutations might confer Imatinib resistance in the AIDS-KS clinical trials [34]. Our sequencing results in KSHV+ve mECK36 tumors as well as the AIDS-trial biopsies, together with other analyzed KS samples [55], showed a wild-type genotype for the PDGFRs and c-kit. Yet, classic Sanger sequencing is probably unsuited to detect mutations that are not occurring in the majority of the cells. Even though further genomic inquiry employing NGS approaches in KS is warranted, if the mutations occur in a small percentage of cells, sequencing coverage may not be able to reveal the existence of mutations. Although in GIST, D842V mutations also confer resistance to Sunitinib [43], in the context of the mECK36 model, Sunitinib was also able to inhibit the D842V mutant tumors, suggesting that concomitant anti-angiogenic targeting might also be able to overcome drug resistance. Importantly, a recent AMC sponsored trial has shown that Sunitinib is compatible with some HAART regimes [41]. These clinical studies and our current results warrant further examination of the use of Sunitinib in AIDS-malignancies and in particular AIDS-KS.

A major breakthrough in targeted therapies for sarcomas is the recent FDA approval of an anti-PDGFRA therapeutic antibody to treat soft tissue sarcomas [56]; which, as we show now for the case of viral sarcomagenesis with KS, are also oncogenically driven by ligand activation of the PDGFRA. The anti-PDGFRA antibody (Lartruvo/ Olaratumab) acts by blocking the interaction of PDGFs with PDGFRA and has been shown to add a significant benefit to patients receiving only doxorubicin, which in its liposomal form (Doxil) is a frontline therapy to AIDS-KS. We postulate that the present results constitute a solid basis for similar clinical studies for testing the benefit of Olaratumab in AIDS-KS patients receiving Doxil. Phospho-PDGFRA IHC in KS tumors would be a biomarker for response and it could also be used to monitor drug activity in the KS tumors.

In summary, our results identify a KSHV-dependent-ligand mediated PDGFR activation pathway that drives KS tumorigenesis. We found that this mechanism operates in human KS and in our mouse model can be targeted therapeutically with NAC, Imatinib/Gleevec, Sunitinib or with dominant-negative genetic intervention to block tumorigenesis. We found that host mutations in this pathway could confer resistance to PDGFR inhibition and should be explored for improving treatment decision options. Taken together, our results identify and validate the PDGFR activation axis as a key vulnerability and therapeutic target in KS (Fig 10).

## Materials and methods

### Cell culture and reagents

mECK36 cells were obtained and cultured as previously described [21]. HEK293T cells were cultured in DMEM medium with standard formulations. Doxycycline was purchased from Clontech (Mountain View, CA); N-acetyl-cysteine (NAC) and Rac1 inhibitor EHT1864 from Sigma-Aldrich (St. Louis, MO); Diphenyleneiodonium Chloride (DPI), AG490 from Calbiochem (EMD Chemicals, Gibbstown, NJ); Groα and PDGF-BB from PeproTech (Rocky Hill, NJ); and Imatinib and Sunitinib from LC Laboratories (Woburn, MA). Antibodies: p-PDGFR (Tyr751), PDGFR, AKT, p-AKT, STAT3 and p-STAT3 and Rac1 were purchased from Cell Signaling Technology (Danvers, MA); PDGFB and LANA from Abcam (Cambridge, MA), PDGFA from Millipore (Billerica, MA); and p-PDGFRA (Y742) from R&D Systems (Minneapolis, MN).

### Phospho-receptor tyrosine kinase (RTK) array

R&D Systems' Mouse Phospho-Receptor Tyrosine Kinase (RTK) Array Kit (Catalog # ARY014) was used to detect levels of phosphorylation of 39 RTKs in mECK36 tumors.

### Mouse growth factor array

RayBiotech C-Series Mouse Growth Factor Antibody Array Kit (Catalog # AAM-GF-3-2), was used to detect 30 Mouse Growth Factors in KSHV+ve mECK36 and KSHV-ve mECK36 tumors.

### ELISA

Soluble Platelet-Derived Growth Factor AA (PDGF-AA) and Platelet-derived growth factor subunit BB (PDGF-BB) were determined using American Research Products ELISA kit (catalog # CSB-E17145m and CSB-EL017709MO).

### Constructs

Tetracycline-inducible vGPCR construct (TET-vGPCR) was cloned into the TRIPZ vector (Open Biosystems) using AgeI and ClaI restriction sites at the 5’ and 3’ ends, respectively. Tetracycline-inducible control vector (TET-RFP) was purchased from Open Biosystems. Expression constructs: vGPCR was cloned into pcDNA3 vector (Invitrogen, Carlsbad, CA) using EcoRI and XhoI restriction sites; and Rac1QL (constitutively active) and Rac1N17 (dominant negative) were cloned into pcDNA3 using KpnI and XhoI restriction sites. PDGFB-Luc construct was cloned from a 450 bp fragment of the human PDGFB promoter and cloned into pGL2 vector (Promega, Madison, WI) using KpnI and HindIII restriction sites as previously described [57].

### Superoxide detection

DHE (Molecular Probes, Carlsbad, CA) staining was done as previously [35]. Briefly, serum-starved cells were incubated with DHE/HBSS at 10 μM for 30 min in a CO_2_ incubator at 37°C (dark conditions), in the presence of growth factors and inhibitors if required. Cells were then washed 3 times with HBSS and images in the red channel were taken using a Zeiss ApoTome Axiovert 200M microscope.

### Luciferase reporter assay

HEK293T cells were transfected using Lipofectamine 2000 (Invitrogen). The renilla luciferase plasmid pRL-TK (Promega, Madison, WI) was used as control for transfection efficiency. Transfected cells were assessed for luciferase activity using the Dual-Luciferase Reporter Assay System (Promega, Madison, WI).

### Real-time quantitative PCR (RT-qPCR)

RNA was isolated with RNeasy Plus Kit (QIAGEN, Valencia, CA) with on-column DNase treatment. 500 ng of RNA was transcribed into cDNA using Reverse Transcription System (Promega, Madison, WI) according to manufacturer's instructions. RT-qPCR was performed using an ABI Prism 7000 Sequence Detection System (Applied Biosystems) with SybrGreen PCR Master Mix (Quanta Biosciences). The following primer sets were used: GAPDH (5'-ACCCAGAAGACTGTGGATGG-3', 5'-CACATTGGGGGTAGGAACAC-3'); LANA (5'-CCTGGAAGTCCCACAGTGTT-3', 5'-AGACACAGGATGGGATGGAG-3'); c-Myc (5'-CAACGTCTTGGAACGTCAGA-3', 5'-TCGTCTGCTTGAATGGACAG-3'); VEGFA (5'-AGCACAGCAGATGTGAATGC-3', 5'-AATGCTTTCTCCGCTCTGAA-3'). In every run, melting curve analysis was performed to verify specificity of products as well as water and–RT controls. Data were analyzed using the ΔΔCT method as previously described [21]. Target gene expression was normalized to GAPDH by taking the difference between CT values for target genes and GAPDH (ΔCT value). These values were then calibrated to the control sample to give the ΔΔCT value. The fold target gene expression is given by the formula: 2^–ΔΔCT^.

### Rac1 activity assay

Serum-starved mECK36 cells were stimulated with PDGF-BB (40 ng/mL) for 10 min. Rac1 inhibitor EHT1864 (50 μM) was added to culture media 10 min before PDGF stimulation. Rac1 pull-down assays were performed using the Rac1 Activation Assay Kit (Millipore, Billerica, MA) following manufacturer’s instructions. Cell lysates were pre-cleared with GST-agarose beads for 20 min at 4°C. After removal of GST-beads, lysates were incubated at 4°C for 60 min with p21-activated kinase 1-binding domain (PBD)-agarose beads, then washed four times with MLB buffer and resuspended in 2x Laemmli buffer and resolved in a 12% SDS-PAGE gel. GST-PBD bound active Rac1 (Rac1-GTP) was detected by immunoblotting using a specific antibody against Rac1. Total Rac1 was detected by immunoblotting in samples from corresponding cell lysates.

### Immunofluorescence staining

Immunostaining was performed as previously described [21]. Briefly, cells and frozen sections from tumors were fixed in 4% paraformaldehyde for 10 min, washed with PBS and permeabilized in 0.2% Triton-X/PBS for 20 min at 4°C. After blocking with 10% FBS/PBS for 60 min, samples were incubated with anti-CD31 (1:200 dilution, R&D Systems, Minneapolis, MN), and anti-VEGFR3 (1:200 dilution, ImClone, New York, NY), anti-phospho-PDGFRA (1:30 dilution, R&D Systems, Minneapolis, MN) antibodies for 1 hour. After PBS washing, samples were incubated with secondary antibody Alexa Fluor 555 for 1 hr (1:500 dilution, Molecular Probes, Carlsbad, CA), washed and mounted with ProLong Gold antifade reagent with DAPI (Molecular Probes, Carlsbad, CA). Images were taken using a Zeiss ApoTome Axiovert 200M microscope. Image quantification was performed with Image (NIH). Immunohistochemistry staining of human KS biopsies were made on discard pathological material.

### Western blotting

Protein concentrations in cell and tumor lysates were quantified using the DC Protein Assay (Bio-Rad, Hercules, CA). 20 μg of proteins were mixed with Laemmli buffer, boiled for 5 min, resolved by SDS-PAGE and transferred to PVDF membranes (Bio-Rad Laboratories, Hercules, CA). Membranes were blocked with 5% nonfat milk/PBS for 1 hr and incubated with primary antibodies (4°C, 16 hrs). After 3 TBS/Tween washes, membranes were incubated with HRP-labeled secondary antibodies (1:10,000 dilution, Promega, Madison, WI) for 1 hr at room temperature. Protein bands were developed using ECL Plus Detection Reagents (GE Healthcare, Piscataway, NJ) and quantified by densitometry with QuantityOne software (Bio-Rad Laboratories, Hercules, CA). To analyze multiple proteins on the same membrane, membranes were washed with Restore PLUS Western Blot Stripping Buffer (Thermo Scientific, Rockford, IL) according to manufacturer’s protocol.

### Animal studies

All mice were housed under pathogen-free conditions. Tumor studies were done in 4- to 6- week-old nude mice obtained from the National Cancer Institute. Tumors were generated by subcutaneous injection of mECK36 cells (3 x 10^5^ cells) as previously described [21]. A group of mice was treated with 40 mM N-acetyl-cysteine (NAC) in drinking water 2 weeks after tumor inoculation (preventive modality) or when tumors were already established (treatment modality)[35] (see Fig legends). This NAC regimen assumes that the steady-state concentration of NAC is 9.2 mM or 1.5 g/L NAC for a 20 g mouse [58]. The control group received regular drinking water without NAC. Imatinib was administered at 150 mg/Kg/twice daily by oral gavage every 12 hrs [59] with a 200 μl volume per animal using 20 gauge gavage needles (Cadence Science, Lake Success, NY). Sunitinib was administered at a 80 mg/Kg/day dose by oral gavage, with a 200 μl volume per animal using 20 gauge gavage needles (Cadence Science). Placebo animals received the same volume of vehicle. Tumor volumes were measured using a caliper every 2 days and calculated using the following formula: [length (mm) × width (mm)^2^× 0.52][57].

### Clinical tissue microarrays analysis

70 skin KS biopsies and controls were analyzed from an ACSR (The AIDS and Cancer Specimen Resource) tissue microarray. Immunohistochemistry of clinical tissue microarrays was performed using a standard protocol of the Immunohistochemistry Laboratory of the Department of Pathology at the University of Miami. Antibody staining of p-PDGFRA from R&D Systems (Minneapolis, MN) was diluted to 1:30 and LANA from Abcam (Cambridge, MA) was diluted 1:40. The expression of p-PDGFRA and LANA were classified into three levels depending on the signal strength.

### Statistical analysis

Statistical significance of the data was determined using two-tailed Student’s t-test. A p-value lower than 0.05 was considered significant. Statistical analysis was performed using Unistat Statistical Package for Microsoft Excel. All values were expressed as means ± standard deviation.

### Ethics statement

The animal experiments have been performed under UM IACUC approval number 13–093. The University of Miami has an Animal Welfare Assurance on file with the Office of Laboratory Animal Welfare (OLAW), National Institutes of Health. Additionally, UM is registered with USDA APHIS. The Council on Accreditation of the Association for Assessment and Accreditation of Laboratory Animal Care (AAALAC International) has continued the University of Miami’s full accreditation.

## Supporting information

S1 Fig PDGF receptor inhibitors Imatinib and Sunitinib inhibit angiogenesis and cell proliferation *in vitro*. (A-B) Proliferation of serum-starved mECK36 cells stimulated with PDGF-BB (40 ng/mL) in the presence of increasing concentrations of Imatinib (A) and Sunitinib (B) for 24 hs. (C-D) VEGF secretion of serum-starved mECK36 cells stimulated with PDGF-BB (40 ng/mL) in the presence of increasing concentrations of Imatinib (C) and Sunitinib (D) was determined by ELISA. (E-F) Phosphorylated (p-PDGFR, Tyr740/751) and total PDGFR levels of serum-starved mECK36 cells stimulated with PDGF-BB (40 ng/mL) in the presence of increasing concentrations of Imatinib (E) and Sunitinib (F) were determined by immunoblotting.
(TIF)

S1 Table Activity of Imatinib and Sunitinib. Inhibitory data is expressed as IC50 in nM concentration.
(TIF)
