# Supplementary figures and images for: KSHV-induced ligand mediated activation of PDGF receptor-alpha drives Kaposi's sarcomagenesis
Source: PLoS Pathog. 2018 Jul 9;14(7):e1007175. doi: 10.1371/journal.ppat.1007175 (PMC6053240; doi:10.1371/journal.ppat.1007175)

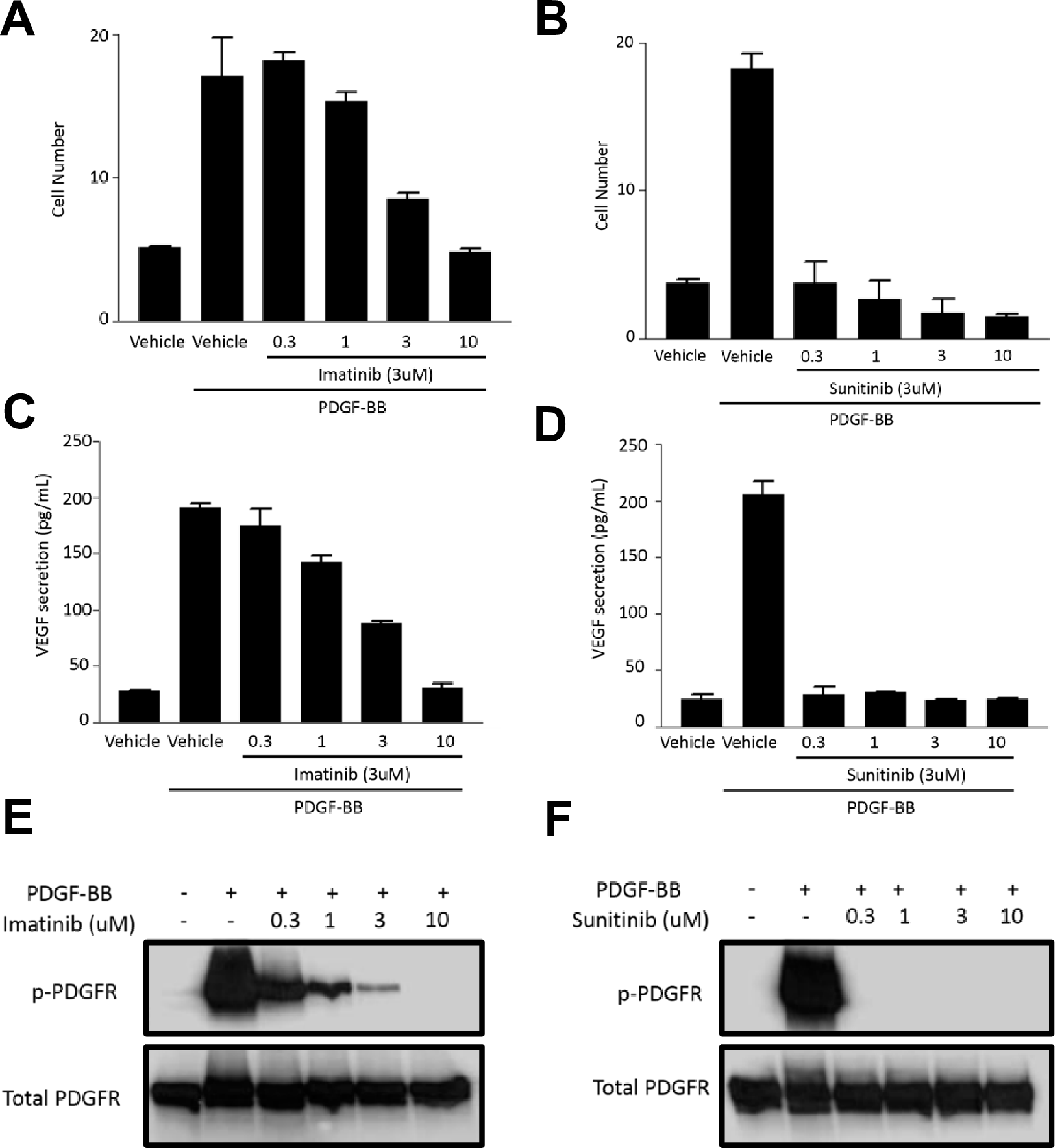

Supplement: S1 Fig — (A-B) Proliferation of serum-starved mECK36 cells stimulated with PDGF-BB (40 ng/mL) in the presence of increasing concentrations of Imatinib (A) and Sunitinib (B) for 24 hs. (C-D) VEGF secretion of serum-starved mECK36 cells stimulated with PDGF-BB (40 ng/mL) in the presence of increasing concentrations of Imatinib (C) and Sunitinib (D) was determined by ELISA. (E-F) Phosphorylated (p-PDGFR, Tyr740/751) and total PDGFR levels of serum-starved mECK36 cells stimulated with PDGF-BB (40 ng/mL) in the presence of increasing concentrations of Imatinib (E) and Sunitinib (F) were determined by immunoblotting. (TIF) [file ppat.1007175.s001.tif]

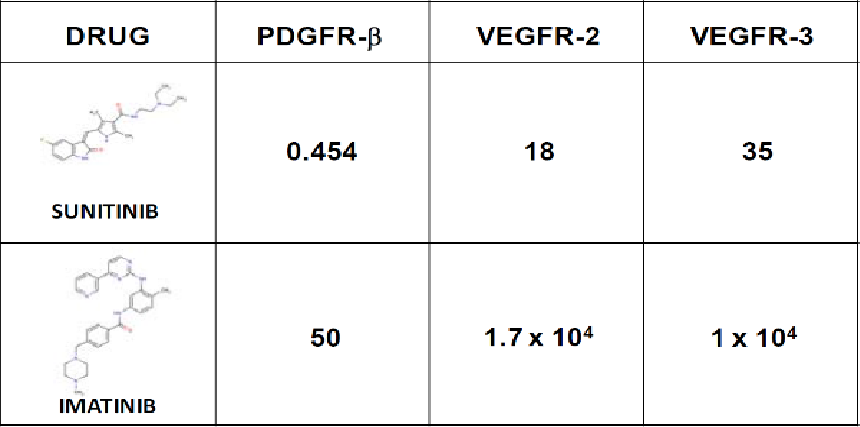

Supplement: S1 Table — Inhibitory data is expressed as IC50 in nM concentration. (TIF) [file ppat.1007175.s002.tif]
